# Supplementary material for: Association between intimate partner violence and male alcohol use and the receipt of perinatal care: Evidence from Nepal demographic and health survey 2011–2016
Source: PLoS One. 2021 Dec 7;16(12):e0259980. doi: 10.1371/journal.pone.0259980 (PMC8651139; doi:10.1371/journal.pone.0259980)

Fig 1: Composition of study sample (Unweighted numbers)

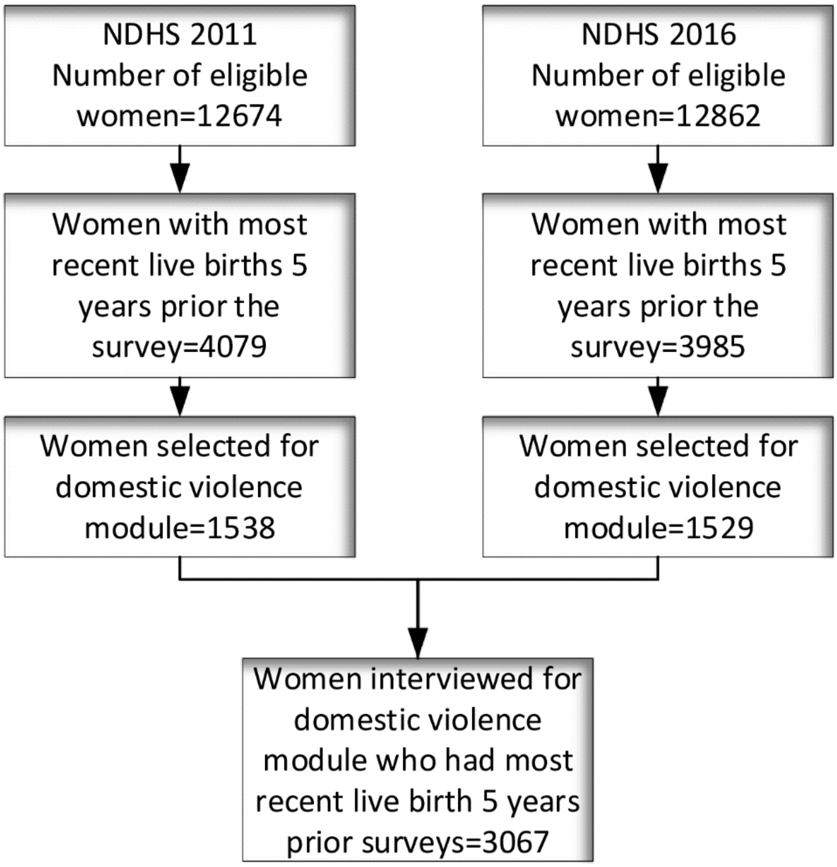

Supplement: Supplementary file 1 [file pone.0259980.s001.docx]
