# Supplementary material for: Association between intimate partner violence and male alcohol use and the receipt of perinatal care: Evidence from Nepal demographic and health survey 2011–2016
Source: PLoS One. 2021 Dec 7;16(12):e0259980. doi: 10.1371/journal.pone.0259980 (PMC8651139; doi:10.1371/journal.pone.0259980)
Supplement: Attachment — Submitted filename: PONE-D-21-11367.docx [file pone.0259980.s002.docx]

This paper used the 2011 and 2016 Nepal Demographic and Health Surveys to look at the association between IPV, male alcohol use and perinatal care. The introduction should be reorganized to flow better and focus on the context of Nepal. The paper can be strengthened by expanding upon why the analysis methods were chosen.

**Major comments:**

Introduction

1. Further description on factors that affect maternal mortality for women in Nepal specifically will be helpful.

2. It would be useful to outline other pathways by which IPV reduces uptake of perinatal care services outside from decision making and poor mental health.

3. Make sure there is consistency in using the term perinatal care and antenatal care.

4. The statement about mothers-in-law and their role seems misplaced. Either expand upon their role and how this relates to IPV or take it out.

Materials & Methods

5.Why are only 4 or more ANC visits considered use of ANC when the recommendation is 8?

6. Clarification and a reason as to why this analysis plan was chosen would strengthen the statistical methods.

**Minor comments:**

Introduction

1. Line 104-105 in the introduction needs a citation.

2. Line 124-127 needs a citation.

3. Line 139 needs a citation.
