# Supplementary material for: Association between intimate partner violence and male alcohol use and the receipt of perinatal care: Evidence from Nepal demographic and health survey 2011–2016
Source: PLoS One. 2021 Dec 7;16(12):e0259980. doi: 10.1371/journal.pone.0259980 (PMC8651139; doi:10.1371/journal.pone.0259980)
Supplement: Attachment — Submitted filename: Review2.docx [file pone.0259980.s004.docx]

1. Though it’s helpful to have the code in the statistical analysis section, it might be better be better placed in a table or annex rather than in the text.

2. What assumptions, if any, were made about the data in order to perform the statistical analysis?

3. Can you further explain how husband alcohol use was quantified (ie, if binary is it that the husband could range from drinking one drink to many in a day)? In the discussion the focus is on alcohol abuse by men, might me good to add in studies that focus on recreational alcohol use as well if the variable of husband alcohol use ranges from recreational use to abuse.

4. Might be important to talk about stigma about reporting IPV and how that could effect your results.
