# Supplementary material for: Association between intimate partner violence and male alcohol use and the receipt of perinatal care: Evidence from Nepal demographic and health survey 2011–2016
Source: PLoS One. 2021 Dec 7;16(12):e0259980. doi: 10.1371/journal.pone.0259980 (PMC8651139; doi:10.1371/journal.pone.0259980)
Supplement: Attachment — Submitted filename: RESPONSE TO REVIEWER 2.docx [file pone.0259980.s005.docx]

**Response to Reviewer 2**

**Comment 1:** Though it’s helpful to have the code in the statistical analysis section, it might be better be better placed in a table or annex rather than in the text.

**Response:** Thank you for your suggestion. However, after reviewing several similar publications we are of the opinion that the preferred way to ensure our study is well understood by the reader is to briefly include the coding in the statistical analysis section. We have also published numerous studies in PLOS ONE as well as in other journals giving the coding information in the statistical analysis section. Please see some similar publications below:

<https://journals.plos.org/plosone/article?id=10.1371/journal.pone.0236435>

<https://journals.plos.org/plosone/article?id=10.1371/journal.pone.0202603>

<https://journals.plos.org/plosone/article?id=10.1371/journal.pone.0223385>

<https://journals.plos.org/plosone/article?id=10.1371/journal.pone.0203278>

<https://www.hindawi.com/journals/bmri/2020/5487164/>

**Comment 2:** What assumptions, if any, were made about the data in order to perform the statistical analysis?

**Response:** Thank you for your comment. However, we do not fully understand your comment. Our study was based on secondary data analysis, and we have provided all necessary information in the statistical analysis section. All limitations to the study have also been stated in the limitation section.

**Comment 3:** Can you further explain how husband alcohol use was quantified (ie, if binary is it that the husband could range from drinking one drink to many in a day)? In the discussion the focus is on alcohol abuse by men, might me good to add in studies that focus on recreational alcohol use as well if the variable of husband alcohol use ranges from recreational use to abuse.

**Response:** In NDHS (2011-2016), women completing the violence module were asked - Does (did) your (last) (husband/partner) drink alcohol? and the results were recorded as binary (YES/NO). Data on quantity of alcohol consumption were not collected in NDHS. Therefore, this study is not able to distinguish if alcohol use by husband was recreational or abuse.

**Comment 4:** Might be important to talk about stigma about reporting IPV and how that could effect your results.

**Response:** Thank you for your comment. In the limitation section, we include "personal or sociocultural perceptions" as an unmeasured co-variate which might have been ruled out in the analysis. Stigma, we believe falls under that category. In addition, further discussion on stigma and how it impact on IPV is beyond the scope of our study.
